# A Perspective on Enzyme Inhibitors from Marine Organisms

**DOI:** 10.3390/md18090431

**Published:** 2020-08-19

**Authors:** Dirk Tischler

**Affiliations:** Microbial Biotechnology, Faculty of Biology and Biotechnology, Ruhr-Universität Bochum, 44780 Bochum, Germany; dirk.tischler@rub.de; Tel.: +49-234-32-22656

**Keywords:** secondary metabolites, functional annotation, structure–function relation, natural products, bioactives, enzyme inhibition, inactivation, marine bacteria, marine fungi, marine sponges

## Abstract

Marine habitats are promising sources for the identification of novel organisms as well as natural products. Still, we lack detailed knowledge on most of the marine biosphere. In the last decade, a number of reports described the potential of identifying novel bioactive compounds or secondary metabolites from marine environments. This is, and will be, a promising source for candidate compounds in pharma research and chemical biology. In recent years, a number of novel techniques were introduced into the field, and it has become easier to actually prospect for natural products, such as enzyme inhibitors. These novel compounds then need to be characterized and evaluated in comparison to well-known representatives. A number of current research projects target the exploitation of marine organisms and thus the corresponding diversity of metabolites. These are often encountered as potential drugs or biological active compounds. Among these, the class of enzyme inhibitors is an important group of compounds. There is room for new discoveries, and some more recent discoveries are highlighted herein.

## 1. Marine Habitats as Sources of Natural Products

More than 70% of the planet Earth’s surface is covered by water, and little is known about the biosphere within these habitats, often designated as marine environment. With respect to its enormous dimension, considering its huge surface area and depths to more than 10,000 m, it is obvious that the diversity of living organisms must be high in marine habitats. Actually, it is assumed that more than 80% of all organisms occur in such marine ecosystems [1]. This provides avenues to new creatures comprising a rich diverse metabolisms and thus novel metabolites can be explored or uncovered.

In the last few years, a number of original articles and reviews have reported on the potential of identifying novel bioactive compounds or secondary metabolites from marine environments, including enzyme inhibitors, which is obviously linked to the tremendous diversity of marine life. In particular, marine bacteria or fungi, cyanobacteria, corals, sponges, algae, and worms, among others, are known to produce a variety of bioactive compounds (including saccharides, polysaccharides, peptides, polyketides, polyphenolic compounds, sterol-like products, alkaloids, quinones, and quinolines, just to name a few compound classes). Often these are produced and excreted as secondary metabolites, as, for example, well-described antimicrobial compounds against Gram-positive bacteria isolated from several marine *Streptomyces* or those of sediments from such ecosystems. These antimicrobial active agents are especially interesting in the context of the still ongoing development of multi-resistant strains, which is critical for our current lifestyle. Anthracimycin [2,3,4] and aplasmomycin [5] are just two representatives to be mentioned that act against Gram-positive bacteria [1]. DNA and RNA biosynthesis are inhibited due to anthracimycin presence [4]. Aplasmomycin is a macrodiolide with a specific inhibitory activity on the futalosine pathway of, for example, *Helicobacter pylori* [5]. Further, it was described to inhibit the growth of several Gram-positive bacteria, including *Mycobacterium* species [6]. Also, 2-alkyl-4-hydroxyquinoline derivatives from *Streptomyces* have bioactive capacities, as some can regulate hyphal growth of *Candida* [7]. These are only a few examples of natural products obtained from marine sources, however, they demonstrate the spectrum of applicability to some extent.

In general, it can be stated that all kinds of bioactivities can be found among the marine-derived natural products, such as those that are antioxidant, anti-Alzheimer, anti-(neuro-)inflammatory, anti-apoptotic, anti-HIV 1, anti-hypertensive, anti-obesity, anti-diabetes, anti-cancer, and radioprotective, as well as those that affect cell-proliferation or show a variety of inhibitory effects.

## 2. Enzyme Inhibitors from Marine Organisms

Enzyme inhibitors are an important field of research, and they allow us to study enzymes and proteins in more detail, as well as to understand pathways and their regulatory network. Often there is an applied aspect in this research, since many enzyme inhibitor surveys are supposed to identify novel bioactive compounds, precursors, or drugs themselves. These efforts can be tackled via various routes, as, for example, by metabolite screening of known producer organisms while altering growth or environment, direct environmental metabolite screening of rich habitats, screening of related strains or species of known producers, sampling complex cultures or habitats, in silico screening of genomes and metagenomes as well as cloning or manipulating efforts of secondary metabolite gene clusters, docking molecules or libraries into target sites, and undoubtedly more methods to be developed.

A marine *Pseudoalteromonas* strain produces a potent α-d-galactosidase with various fields of application or research interests [8]. Therefore, the identification of potential inhibitory molecules and their mode of action is of importance. Here, the sponge *Monanchora pulchra* was chosen to produce or sample directly candidate compounds such as monanchomycalin B, monanchocidin A, and normonanchocidin A. All three compounds belong to the alkaloid family and are described as pentacyclic guanidine alkaloids [9]. They bound to the target galactosidase and showed an irreversible inhibition. The galactosidase was rescued by the use of d-galactose as a competitive inhibitor. A similar study was done with a α-*N*-acetylgalactosaminidase that showed no inhibitory effects of respective alkaloids, which can be explained by the different protein structure. Therefore, the binding mode of the natural compounds from this sponge is directed by the structure of the target protein. Thus, it can be concluded that the sponge metabolites may have therapeutic effects worth studying and even that those can be directed to specific targets. Furthermore, the two studies herein used α-glycosidases from marine bacteria to study the mode of action of novel alkaloid-like molecules.

Recently, a representative of the *Mycosphaerella* genus was probed for novel secondary metabolites [10], and indeed 11 bioactives were determined and partially characterized from this mangrove fungus. Some structures were verified and, in addition, some biological activities were determined. Three of those, namely asperchalasine, epicoccolide B, and epicolactone, showed an inhibition of α-glucosidase. This is relevant for treating diabetes mellitus (type II) and is of high potential, as limited side effects are expected. Some of the remaining compounds, as well as asperchalasine and epicoccolide B, showed antioxidant activity, which is a beneficial finding and product property.

Marine algae have been used for centuries for various purposes, such as food, cosmetics, or a source of bioactive compounds, of course. It is interesting to see that food-derived molecules can be employed as drugs. For example, the algae *Ulva intestinalis* was used to generate a protein hydrolysate containing potential bioactives [11]. Indeed, a pool of angiotensin I-converting enzyme (ACE) inhibitory peptides was determined and characterized. This provides a simple and non-toxic route towards the development of potential antihypertensive compounds. This is in line with other studies on the isolation of bioactive molecules from marine ecosystems for treatment of hypertension [12,13].

Among the polyphenols, the class of phlorotannins is known to show certain properties of interest for pharma research and chemical biology, for example, the ability to treat diabetes or hypertension as well as to inhibit Alzheimer-related proteins [14]. It was recently shown that the brown seaweed *Ecklonia cava* is a prominent source of such phlorotannins [15,16]. Actually, *E. cava* is edible and thus another food-related source of natural products can be used. This seems to be a growing market, as food may serve in the future as a direct means of access to medicine or therapy, although this needs to be explored in more detail. However, the three phlorotannins eckol, dieckol, and 8,8′-bieckol were reported to exhibit inhibitory effects on Alzheimer-related proteins [14]. In silico studies revealed that the hydroxy groups of these compounds allow interaction with the target enzymes, and thus these are potential drug candidates.

Kinases are often used and are therefore validated targets of therapeutic agents. Therefore, many protein kinase inhibitors have already been reported, but further research is being conducted. However, most of these inhibitory molecules are natural products, and many are of marine origin. The latest findings in this field were recently reviewed and cover novel described compounds from 2014 onwards [17]. It seems as though marine habitats and their organisms bear a number of structural diverse molecules that act as kinase inhibitors. Thus, a number of potential candidates for clinical studies can be derived from marine sources.

Inhibitors also play an important role in diet and the management of nutrition. Notably, in fish farms it is important to keep a well-defined balance of nutrients in order to grow fish properly and keep the population healthy. Thus, (anti-)nutritional factors as part of the supplied feed are described in literature. These substances or their metabolites directly affect growth or health. Those with negative effects are called anti-nutritional factors and are often plant-derived and can represent compounds such as phytohormones, protease inhibitors, and lectins, among others [18]. Little is known about anti-nutritional factors from seaweeds, but these are part of the diet given to fish in fish farming operations. A recent study employed *Ulva ohnoi* as model macroalgae in order to study the effects of protease inhibitors on digestives enzymes of marine fish [18]. It was confirmed that *Ulva* produce protease inhibitors that affect fish. It seems as though these protease inhibitors show a reversible and, notably, mixed-type inhibition towards trypsin and chymotrypsin. These effects can be lowered by a thermal pre-treatment of the algae feed prior to feeding fish. Thus, effects by heat-labile anti-nutritional factors can be overcome. It is necessary to study the structure–function relationship of these algae-derived inhibitors in more detail.

## 3. Conclusions

The herein given overview of marine habitats, including their organisms, as a potential source of bioactive compounds, and especially enzyme inhibitors, shows that the field is highly dynamic and still growing in many directions. There are still plenty of putative inhibitors and their modes of action to be discovered. Here, novel methods and combinations of established routes will allow further compounds of interest to be uncovered. In particular, the high-throughput sequencing of genomes and metagenomes of even unculturable organisms will allow novel biosynthetic gene clusters to be discovered, which nowadays can be transferred by means of synthetic toolboxes of molecular biotechnology towards producing organisms. Thus, the production of so far silent or uncovered gene clusters will become more and more useful. Screening and robotics will further develop to assist all these efforts. In addition, we now have bioinformatic tools to pursue molecular dynamic studies, including compound docking, and thus targets and mode of action can be predicted with greater and greater accuracy. Therefore, it can be rationalized that we will see more and more of these products from marine environments.
